# Effects of Probiotic Supplementation on Immune and Inflammatory Markers in Athletes: A Meta-Analysis of Randomized Clinical Trials

**DOI:** 10.3390/medicina58091188

**Published:** 2022-08-31

**Authors:** Yi-Ting Guo, Yu-Ching Peng, Hsin-Yen Yen, Jeng-Cheng Wu, Wen-Hsuan Hou

**Affiliations:** 1 School of Medicine, College of Medicine, Taipei Medical University, Taipei 110, Taiwan; b101106058@tmu.edu.tw (Y.-T.G.); b101106004@tmu.edu.tw (Y.-C.P.); 2 Department of Education, Taipei Medical University Hospital, Taipei 110, Taiwan; 3 School of Gerontology and Long-Term Care, College of Nursing, Taipei Medical University, Taipei 110, Taiwan; kenji@tmu.edu.tw; 4 Department of Urology, School of Medicine, College of Medicine, Taipei Medical University, Taipei 110, Taiwan; 5 TMU Research Center of Urology and Kidney, Taipei Medical University, Taipei 110, Taiwan; 6 Department of Urology, Taipei Medical University Hospital, Taipei 110, Taiwan; 7 Department of Health Promotion and Health Education, College of Education, National Taiwan Normal University, Taipei 106, Taiwan; 8 College of Medicine, National Cheng Kung University, Tainan 701, Taiwan; 9 Department of Physical Medicine and Rehabilitation & Geriatrics and Gerontology, Taipei Medical University Hospital, Taipei 110, Taiwan; 10 Cochrane Taiwan, Taipei 110, Taiwan

**Keywords:** athletes, inflammation-related markers, meta-analysis, probiotics

## Abstract

*Background and Objectives*: Probiotic supplementation can prevent and alleviate gastrointestinal and respiratory tract infections in healthy individuals. Markers released from the site of inflammation are involved in the response to infection or tissue injury. Therefore, we measured the pre-exercise and postexercise levels of inflammation-related markers—tumor necrosis factor (TNF)-α, interleukin (IL)-6, IL-8, IL-10, interferon (IFN)-γ, salivary immunoglobulin A (IgA), IL-1β, IL-2, IL-4, and C-reactive protein (CRP)—in probiotic versus placebo groups to investigate the effects of probiotics on these markers in athletes. Probiotics contained multiple species (e.g., *Bacillus subtilis, Bifidobacterium bifidum*, etc.). *Materials and Methods*: We performed a systematic search for studies published until May 2022 and included nine randomized clinical trials. Reporting followed the Preferred Reporting Items for Systematic Reviews and Meta-analyses guideline. Fixed-effects meta-analyses and sensitivity analyses were performed. Subgroup analyses were conducted on the basis of the period of probiotic intervention and timing of postassessment blood sampling. *Results*: The levels of IFN-γ and salivary IgA exhibited a significant positive change, whereas those of TNF-α and IL-10 demonstrated a negative change in the probiotic group. The subgroup analysis revealed that the probiotic group exhibited significant negative changes in TNF-α and IL-10 levels in the shorter intervention period. For the subgroup based on the timing of postassessment blood sampling, the subgroup whose blood sample collection was delayed to at least the next day of exercise exhibited significant negative changes in their TNF-α and IL-10 levels. The subgroups whose blood samples were collected immediately after exercise demonstrated negative changes in their TNF-α, IL-8, and IL-10 levels. *Conclusions*: Probiotic supplementation resulted in significant positive changes in the IFN-γ and salivary IgA levels and negative changes in the IL-10 and TNF-α levels. No significant changes in the IL-1β, IL-2, IL-4, IL-6, IL-8, or CRP levels were observed after probiotic use in athletes.

## 1. Introduction

Individuals who engage in strenuous exercise are more likely to experience upper respiratory tract and gastrointestinal illness, especially diarrhea, during heavy training and competitions such as a marathon [1,2,3,4]. Strenuous exercise causes immunosuppression by reducing the function of immune cells, thus increasing susceptibility to viral infection [5,6]. Gastrointestinal illness is typically characterized by belching, bloating, flatulence, side stitch, abdominal cramps, vomiting, diarrhea, the urge to defecate during exercise, nausea, and loss of appetite [7,8]. Respiratory illness is often characterized by throat soreness, sneezing, a blocked or runny nose, and cough [8]. Athletes may be more at risk of infection during heavy training [9,10,11], possibly because of the suppression of mucosal immunity, which, in turn, increases susceptibility to gastrointestinal and respiratory illness [2], or alternatively because of the combined effects of small changes in several immune parameters [12]. Therefore, elite athletes are required to reduce the risk of infection and shortly recover from susceptibility to gastrointestinal and respiratory symptoms. Evidence increasingly indicates that probiotic supplementation can prevent and alleviate gastrointestinal and respiratory tract infections (common cold and influenza) in healthy individuals and have an influence on body defense [13,14].

The term probiotic is used for products that deliver the required number of viable cells of bacterial strains that can benefit the health of a host by changing the composition of the host’s gut microbiota [15,16,17,18]. Probiotics mainly serve as supplements [19] containing multiple species (e.g., *Bacillus subtilis*, *Bifidobacterium bifidum*, *Bifidobacterium breve*, *Bifidobacterium lactis*, *Bifidobacterium longum* ES1, *Bifidobacterium animalis* subsp. *lactis*, *Enterococcus faecium* W54, *Lactobacillus acidophilus*, *Lactobacillus brevis* W63, *Lactobacillus casei*, *Lactobacillus fermentum*, *Lactobacillus helveticus*, *Lactococcus lactis*, *Lactobacillus paracasei*, *Lactobacillus plantarum* TWK10, *Lactobacillus rhamnosus* GG, *Lactobacillus salivarius*, and *Streptococcus thermophilus*) and are usually prepared in the form of capsules [8,20,21,22,23], powder sticks [24,25], sachets [26], and fermented drinks [27].

Cytokines, which are small peptides facilitating the influx of lymphocytes, neutrophils, monocytes, and other cells, are released from sites of inflammation and are involved in the response to infections or tissue injury [28,29,30]. Probiotics have been reported to modulate inflammation and systemic immune responses in experimental animals, such as by affecting defense mechanisms and the release of several cytokines (e.g., tumor necrosis factor (TNF)-α and interferon (IFN)-γ) [31,32]. Probiotics can also improve several inflammatory and oxidative stress biomarkers [33]. The balance between proinflammatory and anti-inflammatory cytokines, which regulate immune cell homeostasis, is dynamic and ever-shifting in the human immune system [34,35]. The cytokines initially involved in a cytokine storm include TNF-α, interleukin (IL)-1β, IL-6, and IL-10 [36]. High-intensity long-duration exercise can lead to higher levels of inflammatory mediators, including IL-1β, IL-6, and TNF-α, and thus increase the risk of injury and chronic inflammation [37,38]. IL-2 is considered a key growth and death factor for antigen-activated T lymphocytes [39]. IL-4 is associated with type 2 inflammation, which is related to parasite infection and chronic diseases, including asthma and atopic dermatitis [40]. A systematic review and meta-analysis demonstrated an elevation in IL-1β, IL-8, IL-10, and TNF-α levels; a reduction in IL-2 and IFN-γ levels; and no change in the IL-4 level after long-distance running [41]. However, TNF-α plays a crucial role in several physiological and pathological conditions related to its action in inflammation and leukocyte movement [42]. IL-6 is a cytokine present in circulation during exercise. A study reported that after a person took probiotics, their IL-6 level increased exponentially in response to exercise and declined during the postexercise period [28]. Salivary immunoglobulin A (IgA) as a biomarker is associated with the incidence of infection; its low level or a substantial transitory decline is related to an increase in the incidence of upper respiratory tract diseases [43]. Probiotics increased mucosal salivary IFN-γ, IgA1, and IgA2 levels in healthy adults [44]. However, evidence from clinical trials regarding the effects of probiotic supplementation on immune and inflammatory markers in athletes is lacking. Scholars have reported inconsistent results. Several studies have reported no significant change after probiotic supplementation [20,23,25]. By contrast, some studies demonstrated that the TNF-α level was lower in both sexes after probiotic supplementation [22] and observed a significantly decreased IL-6 level and increased IL-10 level in a probiotic group compared with a placebo group [21]. Moreover, probiotic supplementation attenuated acute exercise-induced changes in both anti-inflammatory and proinflammatory cytokines (IL-6, IL-8, IL-10, IFN-γ, and TNF-α) in male and female athletes [8,45].

This study determined the effect of probiotics on inflammation-related markers (TNF-α, IL-6, IL-8, IL-10, IFN-γ, salivary IgA, IL-1β, IL-2, IL-4, and C-reactive protein (CRP)) in athletes by examining the levels of these markers before and after exercise in probiotic and placebo groups.

## 2. Materials and Methods

### 2.1. Data Sources

The review protocol was prospectively registered on PROSPERO (CRD42022302897), and our findings are reported in accordance with the Preferred Reporting Items for Systematic Reviews and Meta-analyses (PRISMA) guideline. A research librarian systematically searched for relevant studies in PubMed, Cochrane Library, CEPS, and Embase from the inception to 12 May 2022. Citations were managed using Endnote version 20.1 (Clarivate Corp., Philadelphia, PA, USA; London, UK).

### 2.2. Eligibility Criteria

Clinical trials that included healthy human athletes involved in any sport and of any sex, age range, and race and that provided original pre-exercise and postexercise blood data were eligible. We considered interventions involving the administration of probiotics that contained alone or multiple mixed species and were prepared in all forms, including capsules or sticks. Studies that administered to their control group a placebo that was manufactured to be identical to the probiotics in packaging, encapsulation, and taste were eligible for inclusion.

We excluded clinical trials that met one of the following criteria: (1) did not include athletes, (2) were designed as nonparallel randomized clinical trials (RCTs), (3) had only a single arm, (4) did not examine inflammation-related markers, (5) had an intervention period of <14 days, (6) included patients with diseases as the study population, and (7) combined other supplements or medication in their intervention. Furthermore, we excluded studies that measured inflammation-related markers only after the probiotic supplementation.

### 2.3. Data Selection and Extraction

One researcher (YTG) searched for relevant RCTs published in the PubMed, Embase, Cochrane Library, and Chinese Electronic Periodical Services (CEPS) databases from their inception until May 2022. Another researcher (YCP) evaluated the selected RCTs. The researchers were blinded to each other’s decisions. The outcomes were reviewed by two researchers. All retrieved abstracts, studies, and citations were reviewed. The decisions of the two researchers were compared, and if the two reviewers could not reach a consensus, any disagreements were resolved through discussion with a third researcher (WHH).

The two researchers (YTG and YCP) independently extracted data. If data were only presented graphically, values were estimated from figures by using WebPlotDigitizer version 4.5 [46]. Finally, data were analyzed using RevMan 5.4.1 (Cochrane Collaboration, Oxford, UK).

### 2.4. Outcomes

The pre-exercise and postexercise blood levels of inflammation-related markers in the probiotic and placebo groups were measured to determine the effect of probiotics. To perform a meta-analysis, we excluded the outcomes of specific cytokines, which were only measured in one RCT.

### 2.5. Assessment of Risk of Bias

The two reviewers (YTG and YCP) independently determined the risk of bias by using the revised Cochrane risk-of-bias tool for randomized trials, version 2 (RoB 2.0) (Bristol, England) in accordance with the *Cochrane Handbook for Systematic Reviews of Interventions, Version 5.2.12*; this tool measures the potential for bias arising from five domains: the randomization process, deviation from the intended intervention, missing outcome data, outcome measurement, and selection of reported results. Possible responses were “yes”, “probably yes”, “probably no”, “no”, and “no information”. Domains were evaluated as having either low or high risk of bias or some concerns [47]. Assignment or intention to treat was the outcome of interest. Disagreement was resolved through discussion with the third author (WHH).

### 2.6. Statistical Analysis

All analyses were performed using the fixed-effects model with Review Manager version 5.4.1 (Cochrane Collaboration, Oxford, UK), which includes MetaView for presenting graphs and figures. The mean difference and 95% confidence interval (CI) were calculated for each trial and are presented in a forest plot.

To assess heterogeneity, I^2^ statistics were calculated. An I^2^ greater than 50% represents substantial heterogeneity. The potential risk of small-study bias was visually examined by generating funnel plots [48]. Statistical significance was set at a *p* value of <0.05, except for publication bias, where a *p* value of <0.10 was considered. Sensitivity analysis was performed by removing outlier studies—those with CIs that did not overlap with the CI of the pooled effect [49]. If I^2^ was >50%, subgroup analysis was conducted to determine potential factors contributing to the heterogeneity, such as the length of the probiotic intervention (less than 6 weeks vs. more than 6 weeks) and the time of postassessment blood sampling (immediately after exercise vs. delayed to at least the next day of exercise). No further subgroup analysis was performed if an outcome was examined in only two studies. Publication bias was evaluated using Egger’s test. The funnel plot we constructed evaluates the pseudo 95% CI against the standard error of evaluations. Owing to heterogeneity, we used the fixed-effect model because when a conventional funnel plot is used to examine publication bias, the plot is assumed to be inaccurate when the number of studies included in the analysis is small [46].

## 3. Results

### 3.1. Study Selection

**Figure 1:**
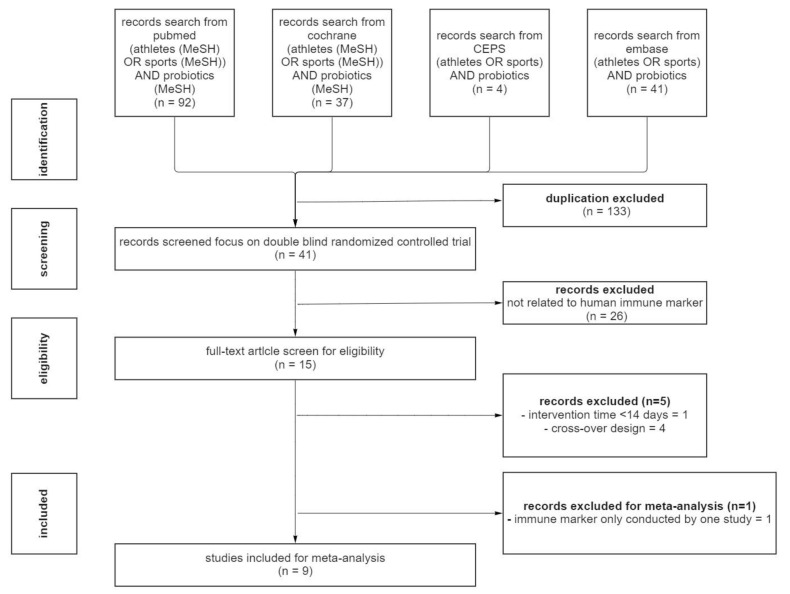
Preferred Reporting Items for Systematic Reviews and Meta-Analysis flowchart of the search strategy.

Figure 1 presents the PRISMA flowchart of the study screening and selection processes used in this research. Through a literature search, we retrieved relevant publications from PubMed (*n* = 92), Cochrane Library (*n* = 37), Embase (*n* = 41), and CEPS (*n* = 4). A total of 41 RCTs were retained after the exclusion of 133 duplicate studies. After the titles and abstracts had been screened, we excluded 26 studies and evaluated the eligibility of the remaining 15 studies. After the full-text assessment, we excluded six trials for several reasons (i.e., intervention time being <14 days, adoption of a crossover design, and examination of inflammation-related markers in only one study). A total of nine studies that met the inclusion criteria were included in this systemic review and meta-analysis.

### 3.2. Characteristics of Included Studies

Table 1 summarizes the characteristics of the nine studies that examined ten inflammation-related markers, namely TNF-α, IL-6, IL-8, IL-10, IFN-γ, salivary IgA, IL-1β, IL-2, IL-4, and CRP. A brief description of their main features is provided in the following sections in compliance with the review strategy.

The nine trials were published between 2011 and 2021, and their sample sizes ranged from 13 to 97. A total of 335 participants were included (170 in the probiotic group and 165 in the placebo group). No difference was noted in age or body mass between the groups. The intervention period did vary somewhat among the studies, ranging from 28 to 90 days.

Five of the selected RCTs lasted 4 weeks (30 days) [21,23,24,25,26], one RCT lasted 8 weeks [27], and three RCTs lasted from 11 to 12 weeks (90 days) [8,20,22]. Sticks, capsules, sachets, or fermented milk were consumed once or twice per day during the supplementation period. Six RCTs used supplementation capsules containing *B. lactis*, and among them, capsules used in three RCTs also contained *B. longum* ES1. The probiotics in each study contained at least one type of *Bifidobacterium* species or *Lactobacillus* species. The control groups were mainly administered sensorially identical placebo capsules, sticks, or sachets containing excipients only without bacteria.

### 3.3. Inflammatory-Related Markers

Of the nine studies, seven reported the TNF-α level, five reported the IL-6 level, four reported the IL-8 level, five reported the IL-10 level, two reported the IFN-γ level, two reported the salivary IgA level, two reported the IL-1β level, two reported the IL-2 level, two reported the IL-4 level, and two reported the CRP level. One study [20] only indicated the differences in these markers after the intervention. Therefore, we determined differences in the levels of these markers by subtracting the preintervention values from the postintervention values.

The timing of the blood sampling in the baseline and postintervention assessments varied. For the baseline assessment, all the studies collected samples prior to the supplementation period with regular exercising. For the postintervention assessment, two studies collected blood samples at 8 a.m. on the 30th day, three studies collected samples after the supplementation period and ensured that participants did not perform strenuous exercise for at least 24 h before sample collection, one study collected samples 1 h after a race, and three studies collected samples immediately after a race.

### 3.4. RoB 2.0 Assessment

**Figure 2:**
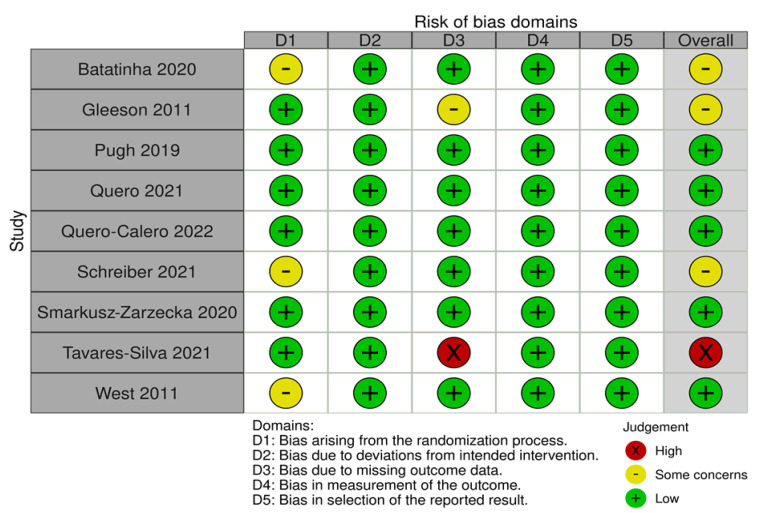
Flowchart of the risk of bias domains.

RoB 2.0 indicated overall high risk for one study, some concerns for three studies, and low risk for five studies for the outcome of inflammation-related markers. Overall, some concerns were concluded for the differences between the probiotic and placebo groups indicating that fat mass was higher in the probiotic group [26], body fat was significantly higher in the placebo group [20], and the white blood cell count was higher in women in the placebo group [8]. Low risk of bias was determined for the blood sampling outcome. We discovered high risk of attrition bias for one study [21] that excluded one participant due to an outlier and had a >20% loss in the follow-up and another study that excluded the data of four participants due to there being insufficient blood volume to enable analyses and had a 31% loss in the follow-up [27]. Because all nine RCTs reported the blinding of assessors, we considered them to have a low risk of detection bias. Studies reporting that the raw data of outcomes were unadjusted were considered to have low risk of reporting bias. Figure 2 presents the risks of bias of all the included studies in the five domains.

### 3.5. Overall Effects

**Figure 3:**
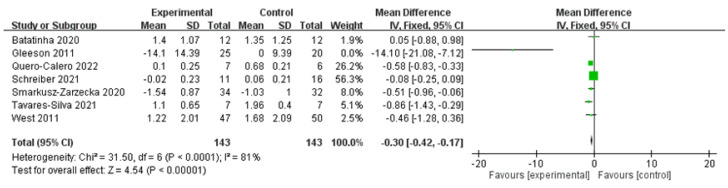
Outcomes for tumor necrosis factor (TNF) −α, indicating that the probiotic group exhibited a significant negative change. An outlier study was noted.

The overall effect size for the TNF-α outcome was −0.30 (95% Cl: −0.42, −0.17, *p* < 0.00001; heterogeneity: chi-square = 31.5, df = 6, *p* < 0.0001, I² = 81%), indicating that the probiotic group exhibited a significant negative change in the TNF-α level compared with the control group (Figure 3). An outlier study was noted [27]. Therefore, a sensitivity analysis was performed by excluding this study for all relevant outcomes.

The effect size for various modes and sites of stimulation is presented in Table 2.

### 3.6. Outcome of TNF-α

**Figure 4:**
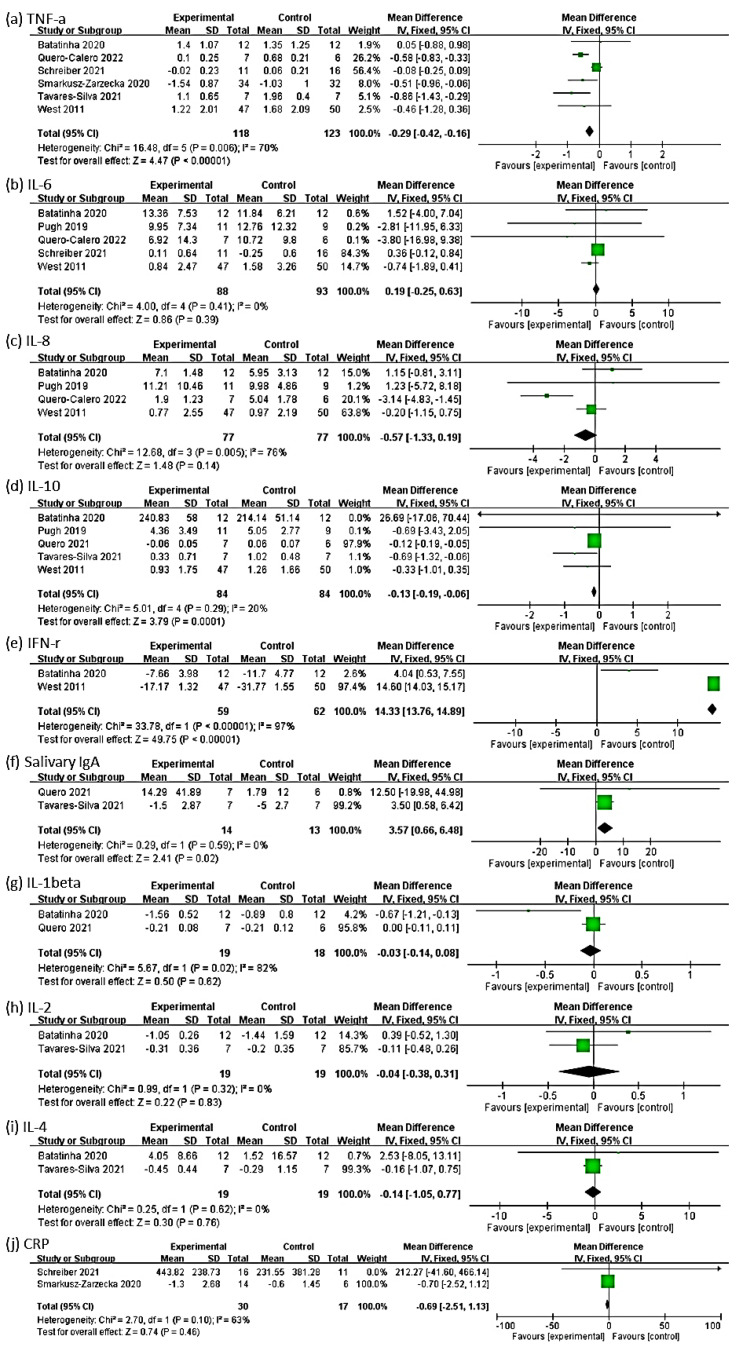
Forest plots of the overall mean effect size for all studies examining the outcomes of (**a**) TNF−α, (**b**) interleukin (IL)−6, (**c**) IL−8, (**d**) IL−10, (**e**) interferon (IFN)−γ, (**f**) salivary immunoglobulin A (IgA), (**g**) IL−1β, (**h**) IL−2, (**i**) IL−4, and (**j**) C−reactive protein (CRP).

The effect size of the remaining six RCTs for the TNF-α outcome was −0.29 (95% Cl: −0.42, −0.16, *p* < 0.00001; heterogeneity: chi-square = 16.48, df = 5, *p* = 0.006, I² = 70%). The probiotic group exhibited a significant negative change in the TNF-α level compared with the control group in the fixed-effect model (Figure 4a). Because an I^2^ value of >50% represents substantial heterogeneity, we performed a subgroup analysis.

**Figure 5:**
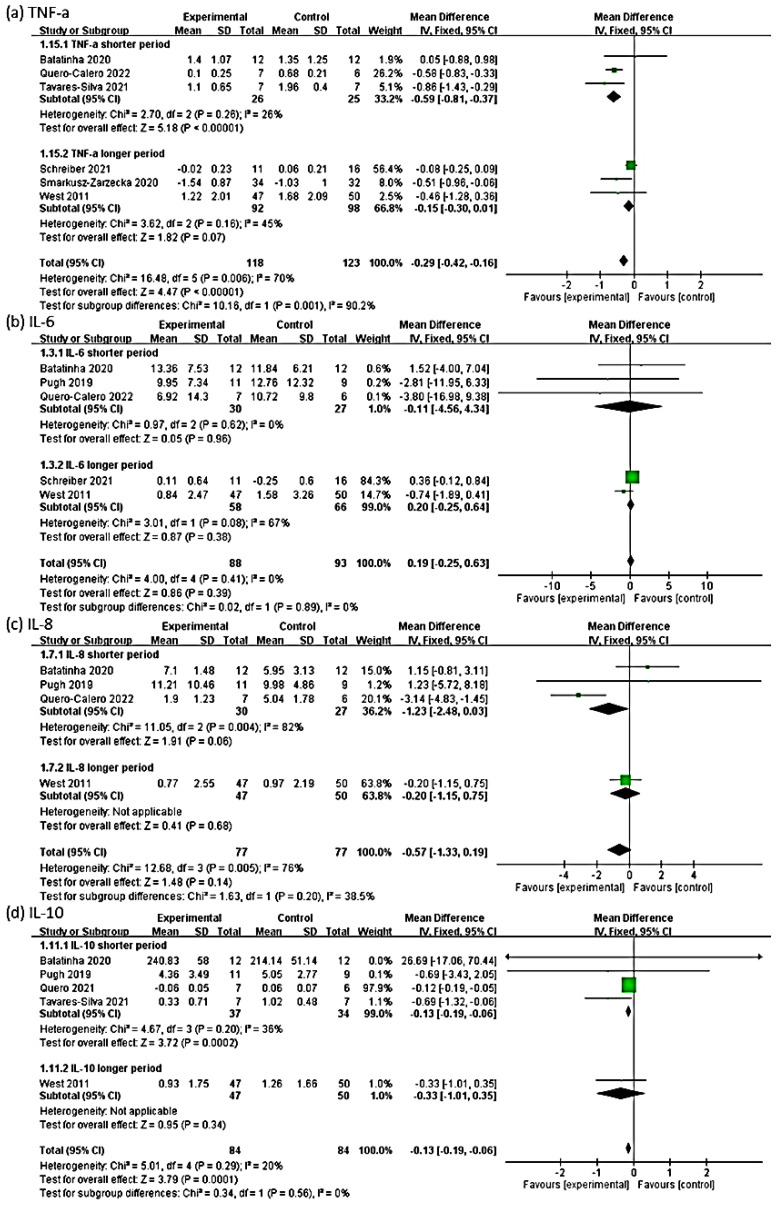
Forest plots of the mean effect size for the subgroups with a shorter and longer intervention period for (**a**) TNF−α, (**b**) IL−6, (**c**) IL−8, and (**d**) IL−10.

**Figure 6:**
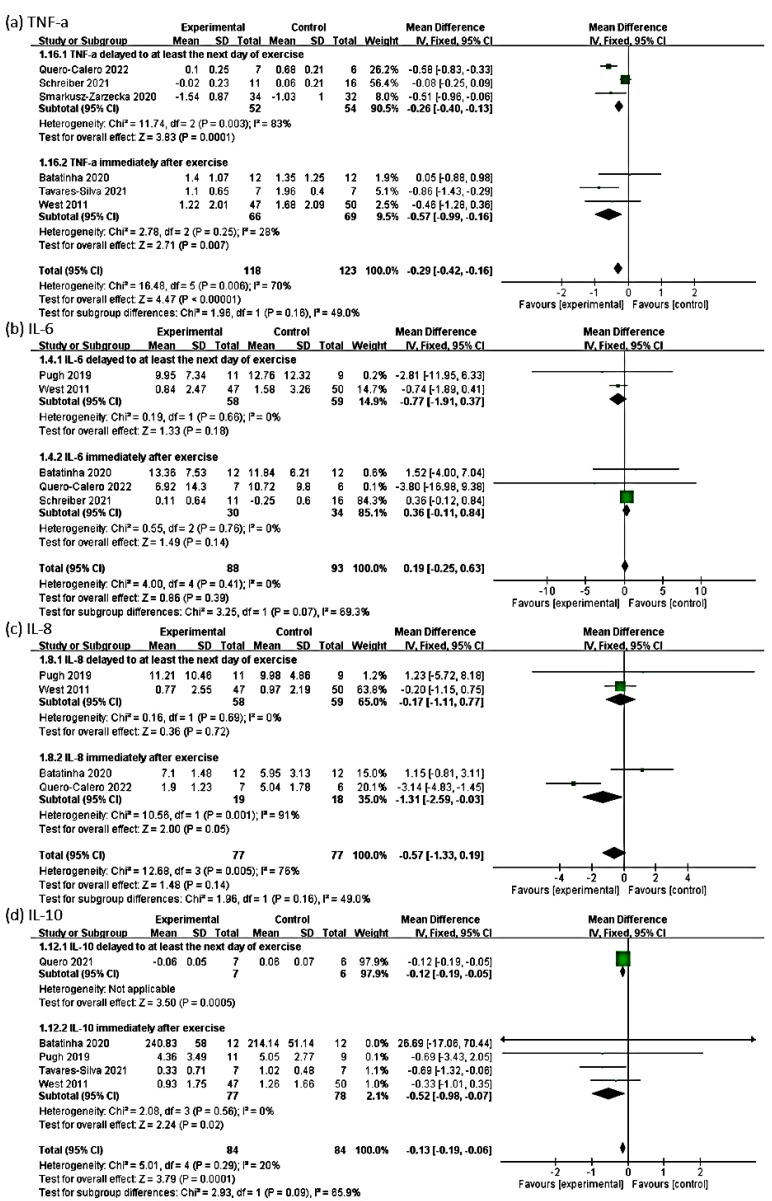
Forest plots of the mean effect size for the subgroups with sample collection delayed to at least the next day of exercise and immediately performed after exercise for (**a**) TNF−α, (**b**) IL−6, (**c**) IL−8, and (**d**) IL−10.

For a shorter intervention period, the probiotic group exhibited a significant negative change in the TNF-α level compared with the control group, with an effect size of −0.59 (95% Cl: −0.81, −0.37, *p* < 0.00001; heterogeneity: chi-square = 2.70, df = 2, *p* = 0.26, I² = 26%). For a longer intervention period, the probiotic group exhibited a significant change in the TNF-α level compared with the control group, with an effect size of −0.15 (95% Cl: −0.30, 0.01, *p* = 0.07; heterogeneity: chi-square = 3.62, df = 2, *p* = 0.16, I² = 45%; Figure 5a). In terms of the timing of postintervention blood sampling, the subgroup whose blood sample collection was delayed to at least the next day of exercise demonstrated a significant negative change in the TNF-α level compared with the control group, with an effect size of −0.26 (95% Cl: −0.40, −0.13, *p* = 0.0001; heterogeneity: chi-square = 11.74, df = 2, *p* = 0.003, I² = 83%). The subgroup whose blood samples were collected immediately after exercise exhibited a significant negative change in the TNF-α level compared with the control group, with an effect size of −0.57 (95% Cl: − 0.99, −0.16, *p* = 0.007; heterogeneity: chi-square = 2.78, df = 2, *p* = 0.25, I² = 28%; Figure 6a).

### 3.7. Outcome of IL-6

The effect size of five RCTs for the outcome of IL-6 was 0.19 (95% Cl: − 0.25, 0.63, *p* = 0.39; heterogeneity: chi-square = 4.00, df = 4, *p* = 0.41, I² = 0%). The probiotic group exhibited no significant change in the IL-6 level compared with the control group (Figure 4b). In the subgroup analysis, for a shorter intervention period, the probiotic group exhibited no significant change in the IL-6 level compared with the control group, with an effect size of −0.11 (95% Cl: −4.56, 4.34, *p* = 0.96; heterogeneity: chi-square = 0.97, df = 2, *p* = 0.62, I² = 0%). For a longer intervention period, the probiotic group exhibited no significant change in the IL-6 level compared with the control group, with an effect size of 0.20 (95% Cl: −0.25, 0.64, *p* = 0.38; heterogeneity: chi-square = 3.01, df = 1, *p* = 0.08, I² = 67%; Figure 5b).

In the subgroup analyses of the timing of postintervention blood sampling, the subgroup whose blood sample collection was delayed to at least the next day of exercise demonstrated no significant change in the IL-6 level compared with the control group, with an effect size of −0.77 (95% Cl: −1.91, 0.37, *p* = 0.18; heterogeneity: chi-square = 0.19, df = 1, *p* = 0.66, I² = 0%). The subgroup whose blood samples were collected immediately after exercise revealed no significant change in the IL-6 level compared with the control group, with an effect size of 0.36 (95% Cl: −0.11, 0.84, *p* = 0.14; heterogeneity: chi-square = 0.55, df = 2, *p* = 0.76, I² = 0%; Figure 6b).

### 3.8. Outcome of IL-8

The effect size of four RCTs for the outcome of IL-8 was −0.57 (95% Cl: −1.33, 0.19, *p* = 0.14; heterogeneity: chi-square = 12.68, df = 3, *p* = 0.01, I² = 76%). The probiotic group exhibited no significant change in the IL-8 level compared with the control group (Figure 4c).

In the subgroup analysis of the intervention period, for a shorter intervention period, the probiotic group exhibited no significant change in the IL-8 level compared with the control group, with an effect size of −1.23 (95% Cl: −2.48, 0.03, *p* = 0.06; heterogeneity: chi-square = 11.05, df = 2, *p* = 0.004, I² = 82%). For a longer intervention period, the probiotic group demonstrated no significant change in the IL-8 level compared with the control group, with an effect size of −0.20 (95% Cl: −1.15, 0.75, *p* = 0.68; Figure 5c).

In the subgroup analysis based on the timing of postintervention blood sampling, the subgroup whose blood sample collection was delayed to at least the next day of exercise exhibited no significant change compared with the control group, with an effect size of −0.17 (95% Cl: −1.11, 0.77, *p* = 0.72; heterogeneity: chi-square = 0.16, df = 1, *p* = 0.69, I² = 0%). The subgroup whose blood samples were collected immediately after exercise exhibited a significant negative change in the IL-8 level compared with the control group, with an effect size of −1.31 (95% Cl: −2.59, −0.04, *p* = 0.05; heterogeneity: chi-square = 10.56, df = 1, *p* = 0.001, I² = 91%; Figure 6c).

### 3.9. Outcome of IL-10

The effect size of five RCTs for the outcome of IL-10 was −0.13 (95% Cl: −0.19, −0.06; *p* = 0.0001, heterogeneity: chi-square = 5.01, df = 4, *p* = 0.29, I² = 20%). The probiotic group exhibited a significant negative change in the IL-10 level compared with the control group (Figure 4d).

In the subgroup analysis based on the intervention period, for a shorter intervention period, the probiotic group exhibited a significant negative change in the IL-10 level compared with the control group, with an effect size of −0.13 (95% Cl: −0.19, −0.06, *p* = 0.0002; heterogeneity: chi-square = 4.67, df = 3, *p* = 0.20, I² = 36%). For a longer intervention period, the probiotic group exhibited no significant change in the IL-10 level compared with the control group, with an effect size of −0.33 (95% Cl: −1.01, 0.35, *p* = 0.34; Figure 5d).

In the subgroup analysis based on the timing of postintervention blood sampling, the subgroup whose blood sample collection was delayed to at least the next day of exercise exhibited a significant negative change in the IL-10 level compared with the control group, with an effect size of −0.12 (95% Cl: −0.19, −0.05, *p* = 0.0005). The subgroup whose blood samples were collected immediately after exercise demonstrated a significant negative change in the IL-10 level compared with the control group in the fixed-effect model, with an effect size of −0.52 (95% Cl: −0.98, −0.07, *p* = 0.02; heterogeneity: chi-square = 2.08, df = 3, *p* = 0.56, I² = 0%; Figure 6d).

### 3.10. Outcome of IFN-γ

The effect size of two RCTs for the outcome of IFN-γ was 14.33 (95% Cl: 13.76, 14.89, *p* < 0.00001; heterogeneity: chi-square = 33.78, df = 1, *p* < 0.00001, I² = 97%). The probiotic group exhibited a significant positive change in the IFN-γ level compared with the control group (Figure 4e).

### 3.11. Outcome of Salivary IgA

The effect size of two RCTs for the outcome of salivary IgA was 3.57 (95% Cl: 0.66, 6.48, *p* = 0.02; heterogeneity: chi-square = 0.29, df = 1, *p* = 0.59, I² = 0%). The probiotic group demonstrated a significant positive change in the salivary IgA level compared with the control group (Figure 4f).

### 3.12. Outcome of IL-1β

The effect size of two RCTs for the outcome of IL-1β was −0.03 (95% Cl: −0.14, 0.08, *p* = 0.62; heterogeneity: chi-square = 5.67, df = 1, *p* = 0.02, I² = 82%). The probiotic group exhibited no significant positive change in the IL-1β level compared with the control group (Figure 4g).

### 3.13. Outcome of IL-2

The effect size of two RCTs for the outcome of IL-2 was −0.04 (95% Cl: −0.38, 0.31, *p* = 0.83; heterogeneity: chi-square = 0.99, df = 1, *p* = 0.32, I² = 0%). The probiotic group exhibited no significant positive change in the IL-2 level compared with the control group (Figure 4h).

### 3.14. Outcome of IL-4

The effect size of two RCTs for the outcome of IL-4 was −0.14 (95% Cl: −1.05, 0.77, *p* = 0.76; heterogeneity: chi-square = 0.25, df = 1, *p* = 0.62, I² = 0%). The probiotic group demonstrated no significant positive change in the IL-4 level compared with the control group (Figure 4i).

### 3.15. Outcome of CRP

The effect size of two RCTs for the outcome of CRP was −0.69 (95% Cl: −2.51, 1.13, *p* = 0.46; heterogeneity: chi-square = 2.70, df = 1, *p* = 0.10, I² = 63%). The probiotic group demonstrated no significant positive change in the CRP level compared with the control group (Figure 4j).

### 3.16. Publication Bias

**Figure 7:**
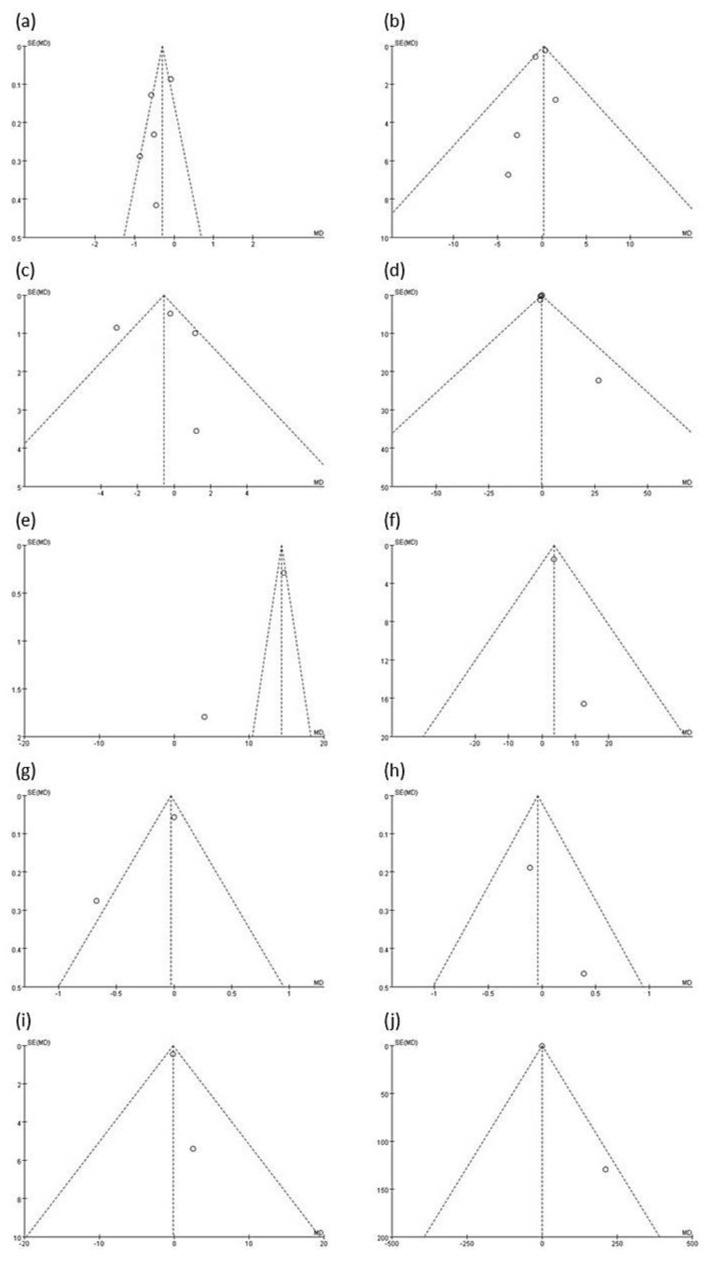
Funnel plots of included studies: (**a**) TNF−α, (**b**) IL−6, (**c**) IL−8, (**d**) IL−10, (**e**) IFN−γ, (**f**) salivary IgA, (**g**) IL−1β, (**h**) IL−2, (**i**) IL−4, and (**j**) CRP.

According to the funnel plots (Figure 7), no heterogeneity was noted for the outcomes of IL-6 (Figure 7b), salivary IgA (Figure 7f), IL-2 (Figure 7h), and IL-4 (Figure 7i) because the included studies appeared to be distributed within two diagonal lines representing their pseudo 95% confidence limits. However, for the outcomes of IL-8 (Figure 7c), IFN-γ (Figure 7e), and IL-1β (Figure 7g), the studies appeared to be distributed beyond two diagonal lines representing heterogeneity [48].

## 4. Discussion

### 4.1. Overall Effect

To the best of our knowledge, this is the first meta-analysis to investigate the effects of probiotic supplementation on the levels of inflammation-related markers, namely IL-1β, IL-2, IL-4, IL-6, IL-8, IL-10, TNF-α, IFN-γ, CRP, and salivary IgA, in athletes.

A study reported that the consumption of a symbiotic bacterium did not affect immune- and inflammation-related markers in athletes [25]. Pugh et al. indicated that the IL-6, IL-8, and IL-10 levels were not significantly different before or after the race between the placebo and probiotic groups, although athletes self-reported lower incidence and severity of gastrointestinal tract symptoms [23]. Schreiber et al. demonstrated that the mean IL-6, TNF-α, and CRP levels were not affected by probiotics [20].

Conversely, some studies have reported the beneficial effects of probiotics. Smarkusz-Zarzecka et al. observed that the TNF-α level was lower in both sexes after probiotic supplementation [22]. Tavares-Silva et al. noted a significant decline in the IL-2 and IL-4 levels 24 h before exercise in the probiotic group compared with the placebo group [21]. West et al. indicated that probiotic supplementation attenuated acute exercise-induced changes in both anti-inflammatory and proinflammatory cytokines (IL-6, IL-8, IL-10, IFN-γ, and TNF-α) in male and female athletes [8].

Moderate activity may enhance immune function to higher than that noted at the sedentary level, whereas intense exercise may cause oxidative stress, muscle damage, inflammation, and immune alteration in elite athletes, leading to upper respiratory tract and gastrointestinal tract illness, especially diarrhea, during heavy training and competitions such as marathons [1,2,3,4,50,51]. In our meta-analysis, we examined the effects of probiotic supplementation on the levels of proinflammatory and anti-inflammatory cytokines in athletes at baseline and after probiotics supplementation. The findings of this meta-analysis including nine studies indicate that not every cytokine participating in the inflammatory reaction had a significantly altered level after probiotic supplementation. No significant difference in the IL-6 level was observed in our meta-analysis. However, we noted significant differences in the IL-8, IL-10, and TNF-α levels.

### 4.2. Proinflammatory Markers: IL-1β, IL-2, IL-4, IL-6, IL-8, TNF-α, and CRP

IL-1β is a key mediator of the inflammatory response [52] and a proinflammatory cytokine that has been implicated in inflammatory conditions [53].

IL-2 plays an immunoregulatory role; it promotes the growth and development of peripheral immune cells in the initiation of the (defensive) immune response and maintains their viability as effector cells [54].

IL-4 is associated with type 2 inflammation and can downregulate IL-1β and TNF-α because of type 1 and type 2’s mutual suppression of each other [55,56].

IL-6 is a key member in the network of cytokines and plays a crucial role in acute inflammation [57]. Moreover, IL-6 exerts proinflammatory effects (e.g., in acute innate responses) and coordinates anti-inflammatory activities essential for the alleviation of inflammation [58].

IL-8 is a chemoattractant cytokine produced by various tissue and blood cells, and it attracts and activates neutrophils in inflammatory regions [59]. In addition to having chemokine properties, IL-8 acts as an angiogenic factor [60].

TNF-α is an inflammatory cytokine produced by macrophages and monocytes during acute inflammation and is responsible for various signaling events within cells, leading to necrosis or apoptosis [42,61].

CRP is a pentameric protein synthesized by the liver, and its level increases in response to inflammation. CRP is primarily induced by IL-6 during the acute phase of an inflammatory or infectious process [62].

Regarding proinflammatory markers, our quantitative analysis demonstrated that probiotic supplementation significantly reduced the TNF-α level but caused no changes in the IL-1β, IL-2, IL-4, IL-6, IL-8, and CRP levels. This result is consistent with that of a previous meta-analysis investigating the effects of probiotic supplementation on normal healthy individuals and reporting a reduction in the TNF-α level but no differences in the IL-1β, IL-4, IL-6, IL-8, and IL-10 levels [63]. Although we did not observe significant changes in the levels of all proinflammatory markers in this study, their levels were lower after probiotic supplementation. In the subgroup analysis based on the timing of the postintervention blood sampling, the subgroup whose blood samples were collected immediately after exercise exhibited a significant decrease in the IL-8 level.

To perform a subgroup analysis on the basis of the period of probiotic intervention, we divided the studies into two groups: those in which athletes received probiotics for less than 6 weeks and for more than 6 weeks. The TNF-α level significantly changed in the shorter period group but not the longer period group, although the *p* value for the longer period group was 0.07, which is close to statistical significance. The current guidelines of the World Gastroenterology Organization indicate that it is generally not possible to state a general dose that is required for probiotics, and the dosage should be based on human studies showing a health benefit [64].

Probiotics may provide benefits by improving mucosal immunity, the inflammatory system, antioxidant capacity, stress reduction, microbiota composition, and the microenvironment in the gastrointestinal tract [65,66].

### 4.3. Anti-Inflammatory Markers: IL-10 and IFN-γ

IL-10 is the most important cytokine with anti-inflammatory properties [67]. In terms of the correlation between the IL-10 level and exercise, the exercise-induced increase in the plasma IL-6 level is followed by increased circulating levels of anti-inflammatory cytokines, such as IL-1ra and IL-10 [35,68].

IFN-γ coordinates a diverse array of cellular programs through transcriptional regulation of immunologically relevant genes [69]. IFN-r is considered an anti-inflammatory cytokine at low concentrations [70].

Our study revealed a reduction in the level of IL-10 but an increase in the level of IFN-γ after probiotic supplementation in athletes. A recent systematic review on this topic reported that exercise duration is the most crucial factor determining the magnitude of the exercise-induced increase in the plasma IL-10 level. However, no significant correlation was noted between the intensity of exercise and change in the IL-10 level [71]. The appearance of IL-10 after eccentric exercise may indicate that IL-10 release is secondary to tissue damage [72]. Thus, the first reason may be the original anti-inflammatory effect of antibiotics. Since probiotics may reduce the levels of proinflammatory markers, they do not further stimulate the production of IL-10.

### 4.4. Salivary IgA

IgA is the dominant immunoglobulin isotype on all mucosal surfaces, where it acts as the first line of defense against microbial invasion [73]. It is observed that oxidative stress is the leading cause of inflammation and may have a negative impact on immune function, so curing of oxidative stress will ultimately suppress the occurrence of inflammation [74]. IgA in sublingual and submandibular secretions is a preferential noninvasive proxy for intestinal immune induction [75]. Studies have reported varying effects of exercise on the IgA level. A meta-analysis conducted in 2021 indicated that physical exercise resulted in a change in the salivary IgA level in athletes; however, this study had risk of bias and very low certainty of the evidence [76].

Our results revealed a significant increase in the salivary IgA level after probiotic supplementation in athletes, indicating that probiotics exert beneficial effects on intestinal immune function. The increase in mucosal immunity due to administration of probiotics can protect against infection from pathogens that penetrate the mucosa [43,77].

Our results demonstrate that probiotics play a role in the anti-inflammatory response; this finding is consistent with those of two previous studies reporting that probiotics exert anti-inflammatory effects in intestinal chronic diseases [78] and can prevent acute upper respiratory tract infections [79]. The current guidelines of the American Gastroenterological Association indicate that in symptomatic children and adults with irritable bowel syndrome, the use of probiotics is recommended only in the context of a clinical trial [64]. The World Gastroenterology Organization concluded that probiotics can treat and prevent acute diarrhea but the mechanisms of action may be strain-specific [64].

### 4.5. Heterogeneity

The results of this study had relatively high heterogeneity; the influential factors were the duration of the intervention, assessment time point, country, probiotic type, and sport type. This study evaluated multiple outcomes on the basis of different intervention and participant types. To reduce the heterogeneity in outcomes between included studies, this study conducted subgroup analyses of the characteristics of supplementation and assessments and analyzed several potential moderators.

### 4.6. Strengths and Limitations

The strength of this meta-analysis is the extensive literature search covering RCTs published over 12 years. Another advantage is that we performed subgroup analyses in relation to several potential moderators. Moreover, we analyzed several types of inflammation-related markers.

This study has some limitations. The first is the quality of the included studies. Three of the nine studies had some concerns of bias and another study had high risk of bias; this may limit the confidence of the conclusion. In one study, fat mass was higher in the probiotic group [26]. In another study, body fat was significantly higher in the control group [20]. In one study, data were excluded due to there being insufficient blood volume to enable analyses and the loss during the follow-up was 31% [27]. In another study, one participant was excluded due to an outlier; this study had over 20% loss during follow-up [21]. Second, heterogeneity still existed regarding different intervention and participant types; the duration of the intervention, assessment time point, country, probiotic type, gender proportion, and sport type affected the evidence of our results. Differences between male and females included the type and intensity of physical activity. We did not review interactions related to the various species used in the supplements and if it was anticipated that there would or could be a synergistic impact on the markers. We mainly focused on probiotic supplementation and excluded studies that combined probiotics with other medications. Additionally, although we investigated the effects of probiotics on athletes, different types of sports were included, which may have resulted in different exercise intensities and thus altered the result. Finally, because the inflammation-related markers we assessed could only serve as a proxy of clinical effectiveness, the actual correlations between inflammatory markers and clinical symptoms, such as gastrointestinal syndromes and upper respiratory tract infection, were unclear.

## 5. Conclusions

This systematic review included nine studies published from 2011 to 2022. The findings of this systematic review and meta-analysis suggest that probiotics result in significant positive changes in the levels of IFN-γ and salivary IgA but negative changes in the levels of IL-10 and TNF-α, which demonstrated that probiotics play a role in the anti-inflammatory response. The levels of IL-1β, IL-2, IL-4, IL-6, and CRP did not exhibit significant changes. Our findings support that probiotics exert anti-inflammatory effects in intestinal chronic diseases and may be strain-specific to treat and prevent acute diarrhea. Future studies investigating the effects of probiotics can use larger samples, examine more types of exercise, and compare more types of probiotics.

## Figures and Tables

**Table 1:** Characteristics of the studies included in the meta-analysis.

Source	Publication Date	Country	Sport Type	Species	Assessed Inflammatory Markers	Probiotic Type	Control (Placebo) Type	Male Female Total	Probiotic Control	Probiotic Control	Probiotics	Baseline Assessment Time	Dose (^16^ CFU)	Frequency	Intervention Time	Post-Intervention Assessment Time
**West, 2011**	2011 April	Australia	Cycling	*L. fermentum*	IL-6, IL-8, IL-10, IFN-γ, TNF-α	Capsule	Capsule	M: 62 F: 35 T: 97	P: 47 C: 50 T: 97	P: 35.2 ± 10.3 C: 36.4 ± 8.9	P: 77.9 ± 8.4 C: 76.9 ± 8.2	Prior to supplement period	1 billion CFU	1 capsule QD	11 weeks	Immediately after a race
**Tavares-Silva, 2021**	2021 April	Brazil	Running	*B. bifidum*, *B. lactis*, *L. acidophilus*, *L. lactis*, *L. paracasei*	IL-2, IL-4, IL-10, TNF-α, salivary IgA	Capsule	Capsule	M: 14 F: 0 T: 14	P: 7 C: 7 T: 14	P: 41.57 ± 3.20 C: 38.28 ± 3.09	P: 71.24 ± 3.55 C: 78.43 ± 8.40	1st day	5 billion CFU	2 g ^20^ QN	30 days	Immediately after a race
**Smarkusz-Zarzecka, 2020**	2020 December	Poland	Running	*B. bifidum*, *B. animalis* subsp. *lactis*, *L. acidophilus*, *L. brevis* W63, *L. casei*, *L. lactis*, *L. salivarius*	CRP, TNF-α	Capsule	Capsule	M: 46 F: 20 T: 66	P: 34 C: 32 T: 66	P: 39.35 ± 8.23 C: 37.62 ± 8.82	P: 72.46 ± 4.62 C: 78.26 ± 6.74	Prior to supplement period	2.5 billion CFU	2 capsules BID	90 days	After the supplement period; physical activity was avoided for at least 24 h before the test
**Schreiber, 2021**	2021 May	Israel	Cycling	*B. subtilis*, *B. lactis*, *B. longum* ES1, *E. faecium* W54, *L. helveticus*	IL-6, ^10^ CRP, TNF-α	Capsule	Capsule	M: 27 F: 0 T: 27	P: 11 C: 16 T: 27	P: 25.9 ± 4.6 C: 29.5 ± 6.2	P: 71.3 ± 8.9 C: 72.0 ± 6.2	1st day	15 billion CFU	1 capsule QD	90 days	90th day; strenuous activity was avoided for at least 24 h
**Quero-Calero, 2022**	2022 March	Spain	Soccer	*B. lactis*, *B. longum* ES1, *L. rhamnosus* GG	IL-6, IL-8, TNF-α	Powder stick	Powder stick	M: 13 F: 0 T: 13	P: 7 C: 6 T: 13	P: 20.6 ± 1.39 C: 21.9 ± 2.77	P: 70.57 ± 6.75 C: 73.95 ± 6.42	1st day	1 billion CFU	1 #QD	30 days	30th day at 8 a.m.
**Quero, 2021**	2021 April	Spain	Soccer	*B. lactis*, *B. longum* ES1, *L. rhamnosus* GG	IL-1β, IL-10, salivary IgA	Powder stick	Powder stick	M: 13 F: 0 T: 13	P: 7 C: 6 T: 13	P: 20.66 ± 1.39 C: 21.9 ± 2.77	P: 70.57 ± 6.75 C: 73.95 ± 6.42	1st day	1 billion CFU	1 #QD	30 days	30th day at 8 a.m.
**Pugh, 2019**	2019 July	UK	Running	*B. bifidum*, *B. animalis* subsp. *lactis*, *L. acidophilus*	IL-6, IL-8, IL-10	Capsule	Capsule	M: 20 F: 4 T: 24	P: 11 C: 9 T: 20	P: 34.8 ± 6.9 C: 36.1 ± 7.5	P: 76.5 ± 9.4 C: 73.5 ± 11.3	4 weeks before the marathon and prior to the supplement period	25 billion CFU	1# ^19^ QDAMPC, 1# on the morning of the race, 1# 2 h before the start	28 days	Immediately after a race
**Gleeson, 2011**	2011 February	UK	Endurance-based activities	*L. casei* Shirota	IL-1β, IL-2, IL-4, IL-6, IL-8, IL-10, IFN-γ, TNF-α, ^9^ salivary IgA	Fermented drink	Identical in color and taste	M: 54 F: 30 T: 84	P: 32 C: 26 T: 58	P: 32 ± 14 C: 25 ± 9	P: 71.2 ± 9.9 C: 71.6 ± 10.7	1st day	6.5 billion CFU	1# ^18^ BID	8 weeks	After 8 weeks and 16 weeks; strenuous activity was avoided for at least 24 h
**Batatinha, 2020**	2020 November	Brazil	Runners	*B. animalis* subsp. *Lactis* *L. acidophilus*	^1^ IL-1β, ^2^ IL-2, ^3^ IL-4, ^4^ IL-6, ^5^ IL-8, ^6^ IL-10, ^7^ IFN-γ, ^8^ TNF-α	Sachets	Sachets	^11^ M: 27 ^12^ F: 0 ^13^ T: 27	^14^ P: 14 ^15^ C: 13 T: 27	P: 35.96 ± 5.81 C: 40.46 ± 7.79	P: 79.30 ± 10.99 C: 72.67 ± 10.20	1st day	20 billion CFU	1# ^17^ QD	30 days	1 h after a race

^1^ IL-1β, interleukin-1 beta; ^2^ IL-2, interleukin-2; ^3^ IL-4, interleukin-4; ^4^ IL-6, interleukin-6; ^5^ IL-8, interleukin-8; ^6^ IL-10, interleukin-10; ^7^ IFN-γ, interferon-gamma; ^8^ TNF-a, tumor necrosis factor-alpha; ^9^ salivary IgA, salivary immunoglobulin A; ^10^ CRP, C-reactive protein; ^11^ M, male; ^12^ F, female; ^13^ T, total; ^14^ P, probiotics; ^15^ C, control; ^16^ CFU, colony-forming unit; ^17^ QD, once a day; ^18^ BID, twice a day; ^19^ QDAMPC, once a day after breakfast; ^20^ QN, once in the night.

**Table 2:** Overall effect of inflammation-related markers.

		Period of Probiotic Intervention		Timing of Postassessment Blood Sampling	
Inflammation-Related Marker	Overall Effect	Shorter Period	Longer Period	Delayed to at Least the Next Day of Exercise	Immediate Assessment after Exercise
TNF-α	−0.29	−0.59	−0.15	−0.26	−0.57
	(−0.42, −0.16)	(−0.81, −0.37)	(−0.30, 0.01)	(−0.40, −0.13)	(−0.99, −0.16)
IL-6	0.19	−0.11	0.20	−0.77	0.36
	(−0.25, 0.63)	(−4.56, 4.34)	(−0.25, 0.64)	(−1.91, 0.37)	(−0.11, 0.84)
IL-8	−0.57	−1.23	−0.20	−0.17	−1.31
	(−1.33, 0.19)	(−2.48, 0.03)	(−1.15, 0.75)	(−1.11, 0.77)	(−2.59, −0.04)
IL-10	−0.1	−0.13	−0.33	−0.12	−0.52
	(−0.19, −0.06)	(−0.19, −0.06)	(−1.01, 0.35)	(−0.19, −0.05)	(−0.98, −0.07)
IFN-γ	14.33				
	(13.76, 14.89)				
IgA	3.57				
	(0.66, 6.48)				
IL-β	−0.03				
	(−0.14, 0.08)				
IL-2	−0.04				
	(−0.38, 0.31)				
IL-4	−0.14				
	(−1.05, 0.77)				
CRP	−0.69				
	(−2.51, 1.13)				
